# A Gas Sensor Based on a Single SnO Micro-Disk

**DOI:** 10.3390/s18103229

**Published:** 2018-09-25

**Authors:** Mateus G. Masteghin, Marcelo O. Orlandi

**Affiliations:** Department of Physical-Chemistry, São Paulo State University, Araraquara, SP 14800-900, Brazil; mgmasteghin@gmail.com

**Keywords:** gas sensor, SnO, semiconductor, single-element device, low power consumption, FIB nanofabrication, sensing mechanism, Debye length

## Abstract

In this study, individual nanofabricated SnO micro-disks, previously shown to exhibit exceptional sensitivity to NO_x_, are investigated to further our understanding of gas sensing mechanisms. The SnO disks presenting different areas and thickness were isolated and electrically connected to metallic electrodes aided by a Dual Beam Microscope (SEM/FIB). While single micro-disk devices were found to exhibit short response and recovery times and low power consumption, large interconnected arrays of micro-disks exhibit much higher sensitivity and selectivity. The source of these differences is discussed based on the gas/solid interaction and transport mechanisms, which showed that thickness plays a major role during the gas sensing of single-devices. The calculated Debye length of the SnO disk in presence of NO_2_ is reported for the first time.

## 1. Introduction

Large-scale combustion of fossil fuels, while well known to contribute to global warming and air pollution, is likely to continue for many decades to satisfy the continued growth in worldwide demands for electrical power, heating and cooling, and high energy density fuels for vehicles. The key byproducts of fossil fuel combustion include gases such as CO and hydrocarbons due to incomplete combustion, NO_2_ that arises from the reaction of atmospheric N_2_ and O_2_ gases at the high temperatures and pressures experienced under combustion, as well as CO_2_ and water vapor. Lean burn vehicles, while more efficient in the consumption of fuel, nevertheless emit much larger quantities of NO_2_ and carbon soot [1]. Considerable progress has been made over the past decades in minimizing such emissions by the introduction of various emission sensors (e.g., zirconia-based electrochemical oxygen sensors) [2,3], catalysts (e.g., three-way catalysts) [4] and traps (e.g., lean NO_x_ traps) [5]. Recent reports have emphasized the continued challenges associated with the cost-effective detection and minimization of NO_x_ emissions from diesel engines [6].

Semiconducting metal oxide (SMO) nanowire-based structures are some of the most studied chemoresistive devices, in which gases adsorbed/chemisorbed on the surface of such materials modulate their conductivity. In terms of SMOs applied for chemoresistive devices, by far the greatest attention has been focused on tin dioxide (SnO_2_), given its robust character and proven sensitivity to many types of gases [7,8]. Recently, other stoichiometries in the Sn-O system have been identified as having exceptional sensor characteristics, including the Sn_3_O_4_ [9,10,11] and SnO [12] systems. In particular, it was demonstrated that a device composed of numerous SnO micro-disks exhibits “Giant Chemo-Resistive” response to NO_2_, as well as high selectivity against other gases [12]. Under exposure to 100 ppm of NO_2_, the SnO micro-disks exhibited a 1000-fold increase in resistance, remarkably high for a material without surface functionalization.

Chemoresistive devices composed of a single SnO_2_ [13,14], ZnO [15] or other 1D structures [16] have received a great deal of recent attention given their miniaturization possibilities, leading to low power consumption. Such devices are considerably more difficult to fabricate than nanowires carpet ones, but they have the advantage of providing simpler interpretation, given well defined current pathways and surface geometries [17].

Lupan et al. [18] reported the dual beam use to produce single-ZnO devices containing nanowires of different diameters, and a sensor signal of 0.020 (equivalent to 1.018 considering R_air_/R_gas_) was obtained when a nanowire with diameter equals to 100 nm was exposed to 10 ppm of H_2_ and simultaneously stimulated by UV-light. Paulowicz et al. [19] showed a dependence between the sensor signal and the morphology for ZnO structures, i.e., the number and geometry of the grain boundaries play a main role in the detection of toxic and explosive gases. Regarding single-SnO_2_ devices, Kuang et al. [20] reported a sensor signal of 32 for 85% of relative humidity in dry air, and Hernandez-Ramirez et al. [21] used a FIB to fabricate a sensor based on SnO_2_ nanowire with less than 40 nm, reporting sensor signals of 24% (corresponding to 1.24) and 13% (alike 1.13) for 1 ppm of NO_2_ and 100 ppm of CO, respectively. Lastly, Tonezzer et al. [22] showed a sensor signal around 10 to 500 ppm of NO_2_ when dealing with a single-SnO_2_ nanowire with 78 nm of diameter.

Moreover, some researchers have emphasized the use of low power devices based on individual nanowire as gas sensors. Hernandez-Ramirez et al. [23] reported the potential use of SnO_2_ nanowire as a CO sensor at 60 °C using 17 mW to reach this temperature in a micro-heater. Prades et al. [24] showed a sensor signal of 22% (equivalent to 1.22) when a single-SnO_2_ was exposed to 0.5 ppm de NO_2_ at about 250 °C reached spending less than 27 µW, in which they called ultralow power consumption gas sensor. Finally, Prades at al. [25] also reported a sensor signal of 1.6 to 5 ppm of NO_2_ and 1.15 to 100 ppm of CO when individual SnO_2_ nanowires worked consuming about 2 mW.

Here we report on the further development of NO_x_ sensors based on the novel use of SnO micro-disks. The main goals of this work were to fabricate sensor devices based on individual SnO micro-disks as the active element and to test their response as gas sensors. By eliminating the potential barriers present at the contacts between the multiple SnO micro-disks we could more directly investigate the intrinsic response of the disks. Moreover, given the small dimensions of the individual disks, the single-element sensors offer a unique potential for the development of low power consumption devices.

## 2. Materials and Methods

The SnO disks were obtained by application of the carbothermal reduction method using SnO_2_ powder (Sigma-Aldrich, São Paulo, Brazil, 99.9% purity) mixed with carbon black (Union Carbide, São Paulo, Brazil, >99% purity). The procedure involved preparing a 1 g mixture of SnO_2_:C in the molar ratio of 1.5:1 that was inserted in a tube furnace maintained at 1135 °C for 75 min under a constant flux of nitrogen (150 cm^3^ min^−1^). Following synthesis, material retrieved from the alumina tube walls was found to contain both SnO disk-like and nanobelt structures that were subsequently separated by sedimentation. Further details were reported previously [12].

**Figure 1:**
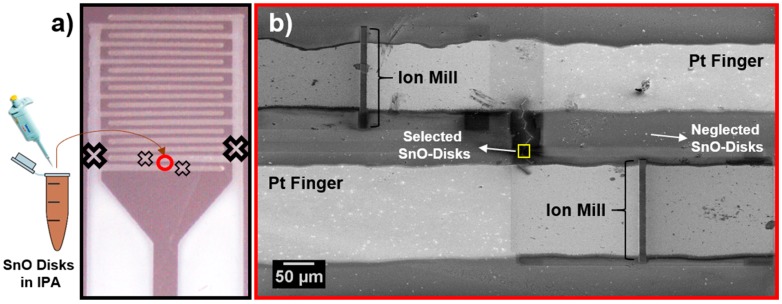
Procedure for device preparation, from the interdigitated electrode deposition to nanofabrication. (**a**) A suspension containing micro-disks deposited onto interdigitated electrodes with crosses representing locations where electrodes were severed by FIB; (**b**) SEM image of the device following nanofabrication process with chosen SnO micro-disk and the Pt- EBID and IBID connecting the sample to the interdigitated fingers. Ion milled region represents cuts made throughout the Pt^0^ on the smaller crossed regions in (**a**). Sensor device within the designated yellow square is shown in Figure 3b.

Figure 1 shows the general procedure followed to obtain single-element sensor devices, whose nanofabrication was performed inside a dual beam microscope (model Helios NanoLab 600i, FEI, Eindhoven, The Netherlands). First, a suspension containing SnO disks was prepared in isopropyl alcohol, dispersed in an ultrasonic bath, and one drop was deposited onto Si/SiO_2_ substrates with interdigitated platinum electrodes (IDE). Then, an isolated SnO disk was identified and connected to the platinum contacts using chemical vapor deposition methods. Finally, the unused Pt electrode fingers of the interdigitated array were electrically isolated from the device using the focused ion beam (black crosses in Figure 1a).

The contacts between the micro-disk and the interdigitated electrodes were prepared with aid of the gas injection system (GIS), by first depositing Pt electrodes at the disk edge by electron beam induced deposition (10 µm long and cross section of 0.25 µm^2^ Pt-EBID, leading to a contact with 800 Ω), and then connecting the disk to the interdigitated fingers using ion beam induced deposition (80 µm long and a cross-section of 1 µm^2^ Pt-IBID, resulting in a contact with somewhat lower resistance [740 Ω]). Two micro-disk devices were prepared, distinguished by their areas: a smaller area disk designated as Device 1 and a larger area disk designated as Device 2, with areas of 1.8 µm^2^ and 2.5 µm^2^, respectively. Finally, the interdigitated fingers contacted by the SEM/FIB Pt-contacts were milled as close as possible to the contacted region (Figure 1b), in order to introduce the minimum electrode interference in sensor response. Details of the SnO micro-disks together with their Pt-EBID and Pt-IBID contacts are shown below.

Gas sensing measurements were carried out by monitoring changes in resistance using a stabilized voltage source (Keithley 6487, Cleveland, OH, USA) applying 0.5 V during cyclic pulse exposures to different concentrations of NO_2_ and CO (20 to 100 ppm) diluted in dry air (baseline gas). Each pulse of analyte gas lasted 10 min followed by 30 min of recovery time, maintaining a constant flux of 100 cm^3^ min^−1^. The measurements were performed at 100 and 200 °C using an external heating chamber with an internal volume of 100 cm^3^. The gas sensor response was evaluated by considering the ratio (R_NO_2__/R_baseline_) for the oxidizing gas and the ratio (R_baseline_/R_CO_) for the reducing gas.

For the small area device, the gas sensor measurements were performed in both oxidizing (NO_2_) and reducing (CO) atmospheres at 100 °C, while for the large area device, gas sensor measurements were carried out for oxidizing gas at 100 and 200 °C, maintaining the same time intervals and flow.

## 3. Results

**Figure 2:**
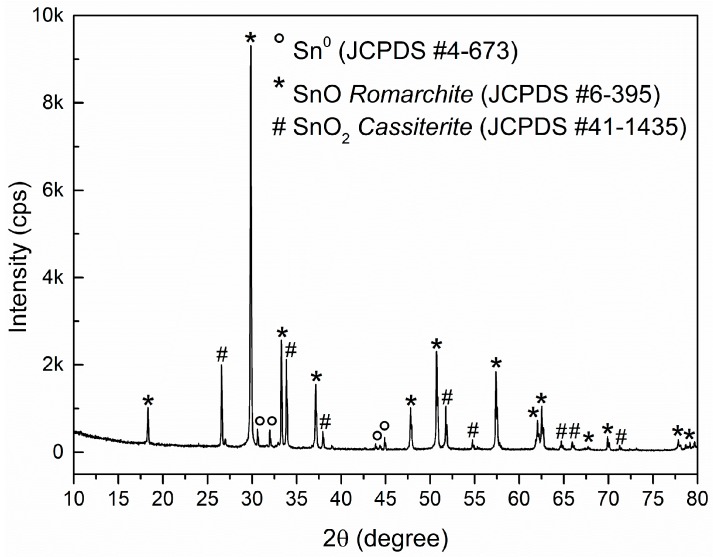
XRD of the SnO micro-disks after synthesis and decantation process. JCPDS used to index the spectra are described in the inset.

Figure 2 presents the XRD of the obtained SnO micro-disks after synthesis and sedimentation process. One can see that the experimental data was indexed using the romarchite phase of the SnO (Space group P4/nmm, litharge-type tetragonal structure, similar to α-PbO), the metallic tin (Sn^0^), and the cassiterite phase of the SnO_2_ (rutile-type tetragonal structure). The predominance of the SnO phase and its high crystallinity matches with the HRTEM and SAED pattern (not shown here), and both Sn^0^ and SnO_2_ phases are related to the well-known disproportionation reaction that SnO undergoes especially in high temperatures and low oxygen pressures, as in inert atmosphere synthesis [26,27].

Thus, considering that a SnO micro-disk was carefully selected inside the Dual Beam microscope, and disk-shaped structures only present the SnO phase [12], one should bear in mind that the reduced Sn^2+^ termination will be the one interacting with the analysis gases.

**Figure 3:**
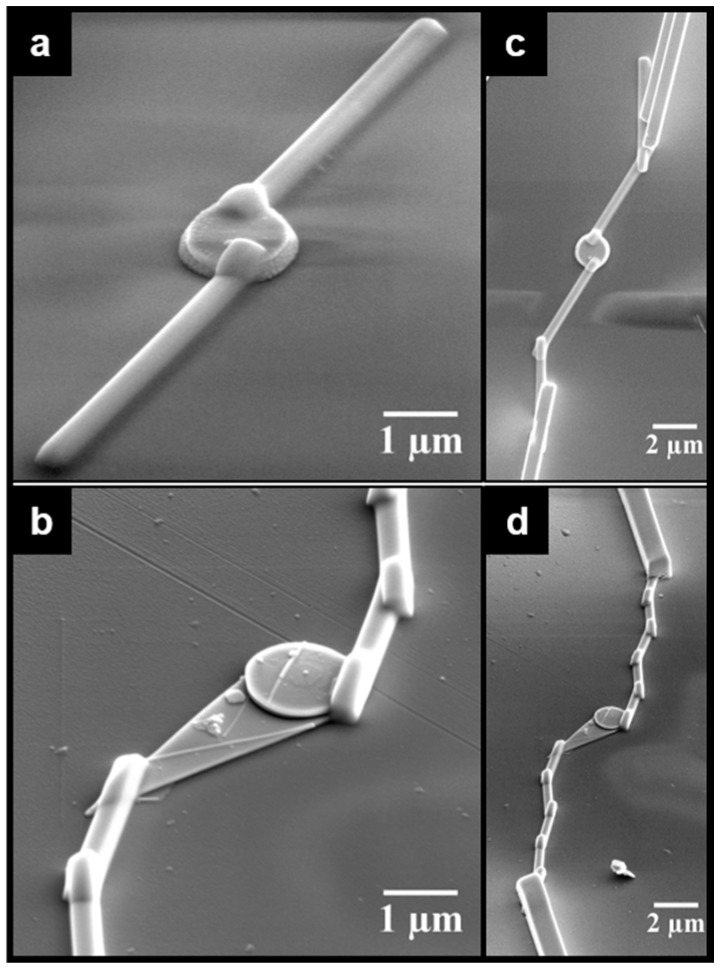
Image of sensor devices made from individual micro-disk. (**a**,**b**) Disks with small and large exposed area, respectively, showing Pt-EBID contacts; (**c**,**d**) The same disks after Pt-IBID electrodes deposition.

Figure 3 shows details of the Pt-EBID and Pt-IBID steps taken to contact the selected SnO micro-disks to the IDEs. Figure 3a,b show high magnification images of the small and large area disks following Pt-EBID, respectively. After contacting the disks by Pt-EBID, they were connected to the platinum IDE using Pt-IBID, as observed in Figure 3c,d. It is possible to observe a clear difference in size of the chosen disks and their different morphologies; the smaller disk (Device 1) presents the most common morphological characteristic of the disks, and the larger area disk (Device 2) exhibiting an extra flat and thinner triangular region, which is related to the growth process of disks.

**Figure 4:**
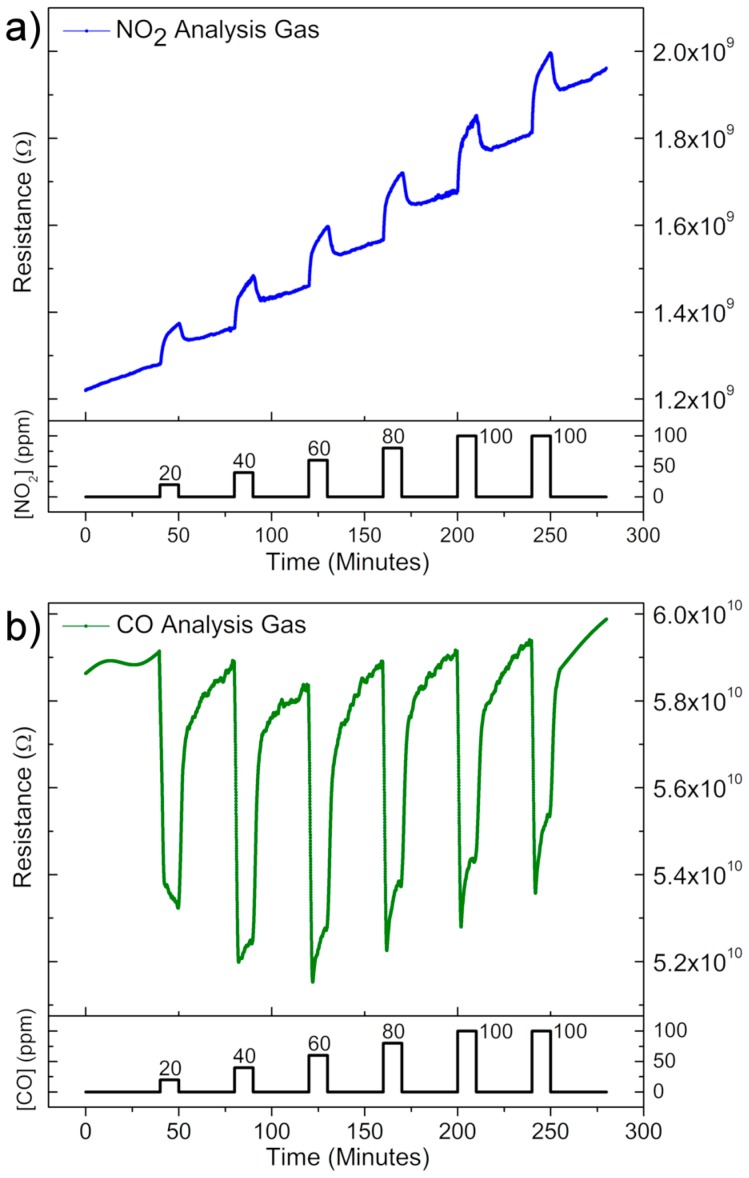
Typical gas sensor measurements for device 1 at different gases concentrations when exposed to (**a**) oxidizing; and (**b**) reducing gas. Results were obtained at 100 °C.

**Figure 5:**
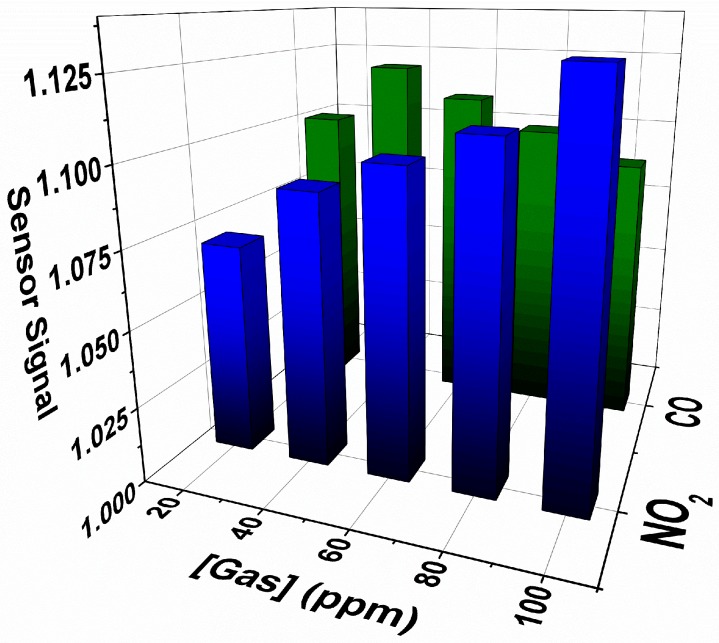
Sensor signal *vs* gas concentration. The signals were calculated through the exponential and linear fittings of the curves shown in Figure 4.

Results presented in Figure 4 for Device 1 clearly reflect n-type semiconductor response, in which the material resistance increases when exposed to the oxidizing gas and the opposite behavior occurs when exposed to the reducing gas, in agreement with previously observed responses obtained with multi-disks SnO sensor [12]. Results also showed that sensor response of the disk increases with increasing NO_2_ concentration, but remains nearly insensitive to variations in CO concentration, as shown in Figure 4 and Figure 5. While single-SnO micro-disk device has lower sensitivity upon exposure to CO at 100 degrees, they work in a more reversible manner based on faster response and quicker baseline recovery.

**Figure 6:**
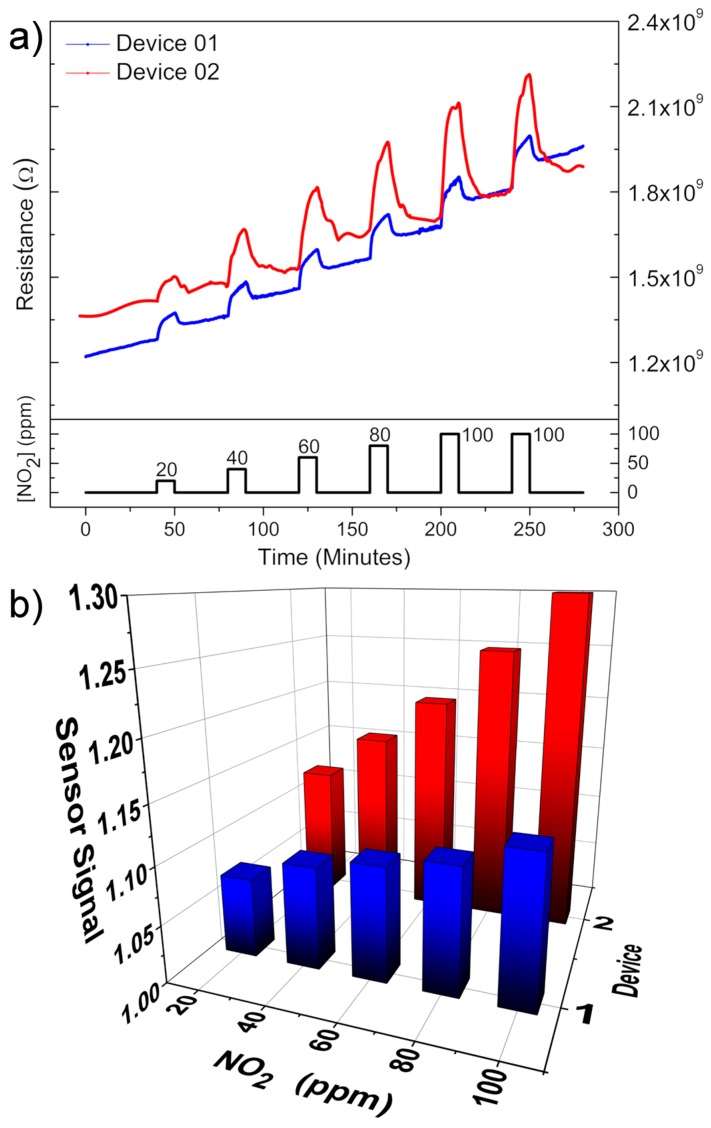
(**a**) Typical gas sensor measurement for different NO_2_ concentration vs time; (**b**) The sensor signal vs gas concentration for the two studied samples. All obtained at 100 °C.

Figure 6 shows the gas sensor response for device 1 (blue curve) and device 2 (red curve) taken at 100 °C for periodic pulses of NO_2_. Although baseline resistance is not constant for the devices, one can observe an increasing sensor response with increasing NO_2_ concentration in both cases (Figure 6a), with the greater sensor response associated, at first, with the larger exposed area device, as observed in Figure 6a,b.

**Figure 7:**
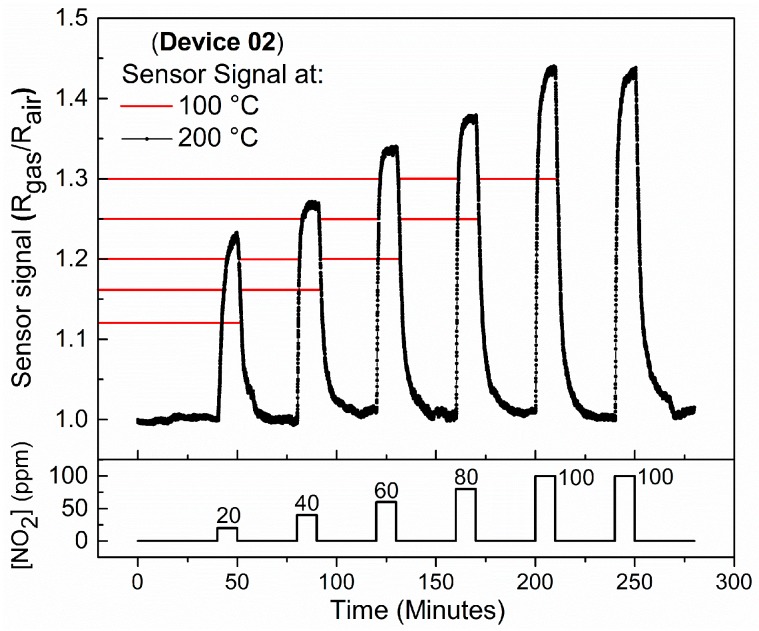
Sensor signal vs. gas concentration obtained at 200 °C. Red lines represent the sensor signals obtained at 100 °C, for comparative purposes.

Figure 7 shows the gas sensor response of Device 2 taken at 200 °C during exposure to NO_2_. It is observed that at this temperature the baseline resistance remains nearly constant throughout the measurement. Moreover, Figure 7 shows that Device 2 presents higher sensor response when exposed to NO_2_ at 200 °C, which is the same temperature that SnO micro-disks carpet devices exhibited the best sensor response [12].

Hence, considering the 0.5 V used to carry on the measurements, and in view of the devices resistance, a power of about 150 pW were dissipated by devices 1 and 2 at 100 °C, and 950 nW was dissipated by Device 2 at 200 °C, when they were exposed to NO_2_. Thus, one can assume that the power lost will be mainly from the heat source, but still should remain only a few dozens of µW due to the small dimension of the structures. Better yet, can be even less power consuming if integrated into hot bodies usually associated with NO_2_ generation.

## 4. Discussion

### 4.1. Temporal Differences in the Sensor Response Curves of Device 1

In order to better understand the obtained sensor response curves in Figure 4, some gas-solid reactions will be elucidated. The first step in the reaction of NO_2_ with SnO is the adsorption of the gas on unoccupied tin surface sites. So, charge transfer can occur via the overlap of the SnO lone pairs (generated by the interaction of the projected anti-bonding molecular orbital (Sn 5s – O 2p_z_)* with the Sn 5p orbital [28,29]), and the NO_2_ molecular orbital (empty π_u_ orbital). After discontinuing the flow of the analyte gas (NO_2_), NO molecules desorb from the surface of the material leaving an adsorbed oxygen ion [30,31]. The charge transfer (CT) and desorption (DES) reactions are summarized by:

(1) $${\mathrm{NO}}_{2\;(\mathrm{gas})}\;+\;{\mathrm{Sn}}^{2+}\;\overset{CT}{\to }\;({\mathrm{Sn}}^{3+}-{\mathrm{NO}}_{{2(\mathrm{ads})}^{-}})\;\overset{DES}{\to }\;({\mathrm{Sn}}^{3+}-O_{{\left(\mathrm{ads}\right)}^{-}})\;+\;{\mathrm{NO}\;}_{\left(\mathrm{gas}\right)}$$

The reactions described in Equation (1) are associated with the differences in temporal response to oxidizing and reducing gases clearly observed when comparing Figure 4a,b. While the baseline resistance drifts continuously higher during the NO_2_ measurements (Figure 4a) it remains nearly constant for the CO measurements (Figure 4b). This behavior is also observed for other single-element sensor devices [32,33] and is related to the known sluggishness associated with the desorption of NO_2_ from semiconducting metal oxide surfaces [34,35], once there is a high binding energy associated with the single and double bonded Sn^2+^-NO_2_ species [36]. This can also be correlated with the difference between recovery times of Device 1 when exposed to NO_2_ and CO, being 3 min and 1 min, respectively.

The reversibility of the gas sensor response to CO can be understood by considering that the prevalent species during adsorption at 100 °C are O_2_^−^ molecules [37,38,39]. Therefore, given a synthetic air baseline, oxygen is naturally adsorbed on the material surface and follows the sequence:

O_2 (gas)_ → O_2 (ads)_ (2)

O_2 (ads)_ + e^−^ → O_2_^−^_(ads)_ (3)

Upon exposure to CO, the following reaction takes place:

2 CO _(gas)_ + O_2_^−^_(ads)_ → 2 CO_2 (gas)_ + 1 e^−^ (4)

One can surmise that the charge transfer reactions described above occur faster than reaction (1), once there is no need for the charge carriers to cross an electric field and reach the surface of the material, i.e., only adsorbed molecules are included in the reactions. This means that the material baseline resistance is easier recovered when the device is exposed to CO pulses than to NO_2_ pulses. This can be associated with the response time values, being approximately 1 min for CO and 7 min for NO_2_.

To explain why the resistance reaches about the same value independently of CO concentration (Figure 4b), giving a rather constant sensor signal (Figure 5), it is essential to understand how single-element sensor devices work. Initially, the crystals are partially depleted due to the adsorption of oxygen species (Equations (2) and (3)), causing the resistance of the material to be an equivalent resistance of the depleted part and the bulk, in which the depleted and non-depleted ratio can be modulated by the presence of analyte gases. Thus, results indicate that only a few ppm of CO is enough to decrease the thickness of the depleted layer in a way that only the bulk resistance plays the role on the SnO micro-disk resistance, causing an almost constant sensor response to CO gas.

### 4.2. Comparison of the Sensor Response for Devices 1 and 2 When Exposed to NO_2_

We previously proposed that the lone pairs present at the (001) plane of SnO are likely the source of the strong observed sensor response to NO_2_. So, it is expected that having higher exposed area will lead to a better sensor response, as observed for device 2 in Figure 6, but it may not be the decisive factor for achieving the 1000-fold change in the resistance, as obtained for the same material in the carpet multi-disks configuration utilized in the previous study [12]. This suggests that the existence of back-to-back potential barriers between the disks may also be an important factor in their large response to NO_2_, similar to recently proposed for single-ZnO structures [19].

Still, the difference in the sensor response shown in Figure 6b (15%) is not close to the difference in the devices exposed area (28%), wherein each side of device 1 has an exposed area of about 1.8 µm^2^ while for device 2 the exposed areas are 2.5 µm^2^.

This divergence can be attributed to the sensing mechanism of individual structures, which should be understood by examining the ratio between the material thickness and the Debye length, which is directly related to the amount of exchanged charges [40]. So, even considering that the total of adsorbed analyte species is larger for Device 2 due to the greater number of free tin ions with exposed lone pairs available to overlap with the NO_2_ molecules, both devices are only partially depleted due to the so-called Weisz limitation where the band bending is limited to about 1 eV as a way to prevent the double-layer potential (number of charged species limited to 10^−5^ to 10^−3^ monolayers) [41,42], causing thickness to play a major role. Equation (5), proposed by Tonezzer et al. [22], shows this dependence mathematically:

Sensor Signal = C _x_ [R/(R − L_D_)]^2^, (5)

where C is a constant depending on the material, R is the radius of the conduction path in the absence of the depleted zone and L_D_ is the Debye Length.

Besides showing the thickness influence on the gas sensor response, Equation (5) can be used to calculate the Debye length of the material. Thus, considering that C and L_D_ values are the same for devices 1 and 2, and thicknesses of 215 nm and 170 nm for devices 1 and 2, respectively, a L_D_ value of approximately 25 nm is calculated for the SnO disks in the presence of 100 ppm of NO_2_ diluted in dry air at 373.15 K, which is similar to values calculated using the semiconducting parameters [12], corresponding to a charge carrier concentration of about 2.8_x_10^21^ m^−3^.

These results indicate the importance of using small size materials to improve the sensor response, as done by Wang et al. [43] using the reported L_D_ value for SnO_2_ nanowires carpet to affirm that the diameter/Debye length ratio has a greater influence on the sensing response than the material exposed area.

However, the area difference can be correlated to the faster response time of Device 2 (5 min) compared to 7 min for Device 1, and longer recovery time of the Device 2 [44]. The recovery times are 3 min and 9 min for devices 1 and 2, respectively. Furthermore, temperature programmed desorption analysis (TPD) showed that just small percentage of the NO_x_ species leaves the surface of the reduced material (SnO) after 2 h [35] corroborating to the high recovery time.

### 4.3. Temperature Influence on the Sensor Response of Device 2 When Exposed to NO_2_

The stability of the baseline in Figure 6 is consistent with the fact that at 200 °C atomic O^−^ species are dominant at material surface, and they have faster reaction kinetics than molecular O_2_^−^ species [37,38,39]. Moreover, at 200 °C both response and recovery times are faster, i.e., 2 and 7 min, respectively. The role of the atomic oxygen ions can be understood based on the reactions [31]:

O^2−^_(ads)_ + O_2 (gas)_ → O_2_^−^_(ads)_ + O^−^_(ads)_ (6)

2 O^−^_(ads)_ → O_2 (gas)_ + 2 e^−^ (7)

2 e^−^ + 2 Sn^3+^ → 2 Sn^2+^ (8)

Figure 7 also presents that device sensor signal for the measurements at 200 °C is higher than the sensor response at 100 °C. We attributed this to the larger depletion layer change induced by the stronger adsorption of the reactive species at the surface of the material coupled with faster charge transfer kinetics [7,12]. Besides, first-principles density functional theory (DFT) calculations showed that adsorbed NO_2_ species have relatively high mobility over the Sn-O surface [36], especially at high temperatures and small area surfaces like in a single-element device, allowing two NO_2_ molecules to react in the material surface generating NO and NO_3_, in which the latter is much more effective to remove electrons from the semiconductor rather than the NO_2_ [36], thereby achieving overall a 1 ppm limit of detection - well below the toxic limits. Moreover, even with NO_3_ having a stronger bonding energy than NO_2_, it helps to stabilize the baseline once there is no evidence of NO_3_ desorbing and leaving oxygen species on the semiconductor surface.

## 5. Conclusions

To summarize, in this work, we have studied for the first time the sensor response of single SnO-disks to oxidizing and reducing gases. Results showed that increasing the exposed surface area to the analyte gas and decreasing the thickness of the micro-disks result in improved device response and that SnO disks exhibit a slightly stronger response to NO_2_ than to CO, especially at high concentrations due to fast CO saturation. Furthermore, these results point to the importance of space charge potential barriers between disks in achieving highest sensor responses and how thickness plays a major role in the sensor signal when dealing with single-element device. The single-element sensor device advantages include the opportunity for device miniaturization, lower power consumption, and low detection limit, although at the expense of reduced sensitivity compared to interconnected micro-disks found in conventionally fabricated devices.
